# Prediction of Subtle Cognitive Decline in Normal Aging: Added Value of Quantitative MRI and PET Imaging

**DOI:** 10.3389/fnagi.2021.664224

**Published:** 2021-07-12

**Authors:** Panteleimon Giannakopoulos, Marie-Louise Montandon, Cristelle Rodriguez, Sven Haller, Valentina Garibotto, François R. Herrmann

**Affiliations:** ^1^Department of Psychiatry, University of Geneva, Geneva, Switzerland; ^2^Medical Direction, Geneva University Hospitals, Geneva, Switzerland; ^3^Department of Rehabilitation and Geriatrics, Geneva University Hospitals and University of Geneva, Geneva, Switzerland; ^4^Department of Neuroradiology, Faculty of Medicine of the University of Geneva, Geneva, Switzerland; ^5^CIRD—Centre d’Imagerie Rive Droite, Geneva, Switzerland; ^6^Department of Surgical Sciences, Radiology, Uppsala University, Uppsala, Sweden; ^7^Department of Nuclear Medicine and Molecular Imaging, Diagnostic Department, Geneva University Hospitals and University of Geneva, Geneva, Switzerland

**Keywords:** amyloid load, atrophy, brain metabolism, cognition, normal aging, quantitative imaging

## Abstract

Quantitative imaging processing tools have been proposed to improve clinic-radiological correlations but their added value at the initial stages of cognitive decline is still a matter of debate. We performed a longitudinal study in 90 community-dwelling elders with three neuropsychological assessments during a 4.5 year follow-up period, and visual assessment of medial temporal atrophy (MTA), white matter hyperintensities, cortical microbleeds (CMB) as well as amyloid positivity, and presence of abnormal FDG-PET patterns. Quantitative imaging data concerned ROI analysis of MRI volume, amyloid burden, and FDG-PET metabolism in several AD-signature areas. Multiple regression models, likelihood-ratio tests, and areas under the receiver operating characteristic curve (AUC) were used to compare quantitative imaging markers to visual inspection. The presence of more or equal to four CMB at inclusion and slight atrophy of the right MTL at follow-up were the only parameters to be independently related to the worst cognitive score explaining 6% of its variance. This percentage increased to 24.5% when the ROI-defined volume loss in the posterior cingulate cortex, baseline hippocampus volume, and MTL metabolism were also considered. When binary classification of cognition was made, the area under the ROC curve increased from 0.69 for the qualitative to 0.79 for the mixed imaging model. Our data reveal that the inclusion of quantitative imaging data significantly increases the prediction of cognitive changes in elderly controls compared to the single consideration of visual inspection.

## Introduction

In recent years, several imaging modalities have been proposed to follow up the evolution of the neurodegenerative process and predict the initial phases of cognitive decrement prior to mild cognitive impairment (MCI). Among them, amyloid and tau imaging ligands were used to assess directly Alzheimer’s’s disease (AD) lesion burden in the human brain, whereas MRI volumetry and FDG-PET metabolic data in AD signature cortical areas are thought to explore downstream cell events leading to neuronal loss. However, the deterioration of cognitive performances in old age is a multifactorial phenomenon involving education attainment, physical condition, and health-related behaviors. In relation with these two latter parameters, vascular burden, mainly cortical microbleeds (CMB) and white matter hyperintensities (WMH) is thought to represent a major determinant of cognitive decrement in old age (Visser et al., 2002; Cavallin et al., 2012; Meier et al., 2014; Petersen et al., 2016; Daugherty and Raz, 2017; Hirsiger et al., 2017; Ten Kate et al., 2017; Vanhoenacker et al., 2017; Bauer et al., 2018; Aljondi et al., 2020). In routine clinical settings, the assessment of these parameters is usually made by visual inspection that reveals only information visible at human eye resolution and depends on the degree of expertise of the rater. Quantitative imaging processing tools have been proposed to support the in-vivo assessment of brain PET and MRI studies including summary metrics of uptake values in standard regions and voxel-wise analyses for PET imaging. While for amyloid PET imaging the summary metric of standardized uptake value ratio (SUVR) of a set of cortical regions divided by a reference region is most commonly used, a voxel-wise comparison with a reference dataset is often performed for FDG-PET, even if summary metrics also exist, such as Meta-ROI (Landau et al., 2010), hypometabolic convergence index (HCI; Chen et al., 2011) and PMOD Alzheimer’s discrimination analysis tool (PALZ; Herholz et al., 2002). In respect to MRI studies, the most widely morphometric analysis methods are volume-based gray matter measures such as voxel-based morphometry by FSL^1^ and surface-based cortical thickness measures by FreeSurfer^2^. The former has the advantage to be closer to the FDG-PET and amyloid PET analyses which do not use a cortical thickness approach. Moreover and in contrast to FreeSurfer, VBM control for head volume biases. Early studies showed a very good to excellent correlation between these measures and visual rating (Vandenberghe et al., 2013; Thurfjell et al., 2014; Mountz et al., 2015). The added value of quantitative protocols compared to the simple visual inspection is still a matter of debate. Some studies indicated that voxel-based hippocampal atrophy data allowed for better discrimination of MCI cases compared to visual rating scores (Li et al., 2019). In contrast, a unified comprehensive visual rating scale has been shown to have a better correlation with age than voxel-based MRI regional analysis (Jang et al., 2015). A 3D stereotactic surface projection was superior to visual SPECT analysis in discriminating very early AD cases (Imabayashi et al., 2004). In contrast, PET studies with support vector machines did not demonstrate a clear advantage of this method compared to visual inspection in the classification of MCI converters (Vandenberghe et al., 2013; Jang et al., 2015; Morris et al., 2016). Moreover, averaged visual ratings and global cortical standard uptake value ratios (SUVRs) disagreed on their classification in almost 20% of ^18^F flutemetamol scans (Mountz et al., 2015). Most of these studies were cross-sectional, explored one imaging modality, and mainly included MCI and AD cases. In the last years, we have focused our research on the very early phases of cognitive decrement that are still compatible with normal aging (Haller et al., 2019, 2020; Montandon et al., 2020b). In a recent study using qualitative MRI markers in the context of a 4.5-year longitudinal study in older community-dwellers, we reported that the accumulation of four CMB or more at baseline as well as increase of visually assessed right MTA at follow-up (but not Fazekas score, amyloid positivity or abnormal FDG-PET patterns) were the only qualitative imaging markers to be independently associated with progressive deterioration of cognitive performance within the age-adjusted norms (Montandon et al., 2020b). In order to examine whether quantitative imaging markers improved the clinico-radiological correlations in elderly controls, we performed a longitudinal study in 90 community-dwelling elders from the same cohort including: (a) visual assessment of medial temporal atrophy (MTA), Fazekas score of white matter hyperintensities (WMH), and CMB (at inclusion and last follow-up), (b) ROI analysis of MRI volume in AD-signature areas (as well as volume loss at last follow-up), and (c) visual inspection of amyloid positivity and cortical SUVRs of amyloid burden, as well as visual and ROI analysis of FDG-PET metabolism in AD-signature areas at last follow-up.

## Materials and Methods

### Study Population

The study was performed according to the declaration of Helsinki and approved by the ethical committee of Geneva, Switzerland. All participants gave written informed consent. All of the cases were recruited via advertisements in local newspapers and media. The cohort included 526 elderly Caucasian white individuals living in Geneva and the Lausanne catchment area, recruited in the context of the Geneva study of brain aging that was funded by the Swiss National Foundation for Research. Due to the need for excellent French knowledge (in order to participate in detailed neuropsychological testing) the vast majority of the cohort were Swiss (or born in French-speaking European countries, 92%; Xekardaki et al., 2015; Zanchi et al., 2017a; Van Der Thiel et al., 2018). Education was assessed as an ordinal variable as a function of the formal years of training (<9: obligatory, 9–12: high school, >12: university). Exclusion criteria included psychiatric or neurologic disorders, sustained head injury, history of major medical disorders (neoplasm or cardiac illness), alcohol or drug abuse, regular use of neuroleptics, antidepressants, or psychostimulants, and contraindications to PET or MRI scans. To control for the confounding effect of vascular pathology on MRI findings, individuals with subtle cardiovascular symptoms, hypertension (non-treated), and a history of stroke, or transient ischemic episodes were also excluded from the present study. We included in the current investigation those individuals who met the following inclusion criteria: (i) three neurocognitive assessments (see below) at baseline, 18 months, and 54 months, (ii) structural brain MRI at baseline and 54 months, (iii) brain amyloid and FDG-PET at 54 months, and (iv) APOE status at baseline. Our sample included 90 elderly individuals (mean age at inclusion 79.2 ± 3.7, range 64–88 years, 96 (61.1%) females; Table 1).

**Table 1:** Demographic, clinical, and imaging data by education attainment level in the present series.

	Education (year)				
	<9	9–12	>12	Total	*P*-Values
N	13	41	36	90
MRI			
Number of microbleeds					0.091
0	7 (53.8%)	24 (58.5%)	27 (75.0%)	58 (64.4%)
1	2 (15.4%)	9 (22.0%)	4 (11.1%)	15 (16.7%)
2+	4 (30.8%)	8 (19.5%)	5 (13.9%)	17 (18.9%)
Fazekas score					0.390
Absent	2 (15.4%)	18 (43.9%)	15 (41.7%)	35 (38.9%)
Mild	8 (61.5%)	15 (36.6%)	13 (36.1%)	36 (40.0%)
Moderate	2 (15.4%)	7 (17.1%)	6 (16.7%)	15 (16.7%)
Severe	1 (7.7%)	1 (2.4%)	2 (5.6%)	4 (4.4%)
MTA right					0.439
No atrophy	6 (46.2%)	8 (19.5%)	9 (25.0%)	23 (25.6%)
Only widening of choroid fissure	4 (30.8%)	20 (48.8%)	18 (50.0%)	42 (46.7%)
Also widening of temporal horn of lat. ventricle	2 (15.4%)	12 (29.3%)	9 (25.0%)	23 (25.6%)
Moderate loss of hippocampal volume	1 (7.7%)	1 (2.4%)	0 (0.0%)	2 (2.2%)
MTA left					0.353
No atrophy	7 (53.8%)	6 (14.6%)	9 (25.0%)	22 (24.4%)
Only widening of choroid fissure	3 (23.1%)	27 (65.9%)	21 (58.3%)	51 (56.7%)
Also widening of temporal horn of lat. ventricle	2 (15.4%)	7 (17.1%)	6 (16.7%)	15 (16.7%)
Moderate loss of hippocampal volume	1 (7.7%)	1 (2.4%)	0 (0.0%)	2 (2.2%)
Posterior cingulate MRI volume change *100	−1.88 ± 1.70	−1.80 ± 1.71	−1.55 ± 1.77	−1.71 ± 1.72	0.764
Baseline hippocampus MRI volume	0.4 ± 0.0	0.4 ± 0.0	0.4 ± 0.0	0.4 ± 0.0	0.527
PET
Amy PET positive	1 (7.7%)	12 (29.3%)	9 (25.0%)	22 (24.4%)	0.495
FDG PET abnormal	2 (15.4%)	12 (29.3%)	8 (22.2%)	22 (24.4%)	0.551
Vermis FDG-PET	1618.7 ± 186.0	1577.8 ± 161.6	1472.8 ± 257.8	1541.7 ± 214.3	0.036
Mesial temporal cortex FDG-PET	1791.5 ± 169.7	1739.5 ± 163.7	1624.4 ± 266.5	1700.9 ± 219.3	0.018
Superior temporal cortex FDG-PET	1781.7 ± 143.5	1700.8 ± 167.5	1608.4 ± 265.3	1675.5 ± 216.4	0.026

### Neurocognitive Assessment

At baseline, all individuals were evaluated with a detailed neuropsychological battery, including the Mini-Mental State Examination (MMSE; Folstein et al., 1975), the Hospital Anxiety and Depression Scale (HAD; Zigmond and Snaith, 1983), and the Lawton Instrumental Activities of Daily Living (IADL; Barberger-Gateau et al., 1992). The cognitive assessment included: (a) attention (Digit-Symbol-Coding, Wechsler, 1997; Trail Making Test A, Reitan, 1958), (b) working memory [verbal: Digit Span Forward, Wechsler, 1955; visuospatial: Visual Memory Span (Corsi), Milner, 1971], (c) episodic memory (verbal: RI-48 Cued Recall Test, Buschke et al., 1997), visual: Shapes Test (Baddley et al., 1994), (d) executive functions (Trail Making Test B, Reitan, 1958; Wisconsin Card Sorting Test and Phonemic Verbal Fluency Test, Heaton et al., 1993), (e) language (Boston Naming, Kaplan et al., 1983), (f) visual gnosis (Ghent Overlapping Figures), (g) praxis: ideomotor (Schnider et al., 1997), reflexive (Poeck, 1985), and constructional [Consortium to Establish a Registry for Alzheimer’s Disease (CERAD), Figures copy, Welsh et al., 1994]. All individuals were also evaluated with the Clinical Dementia Rating scale (CDR; Hughes et al., 1982). The exclusion of MCI cases was based on the criteria of Petersen et al. (Petersen et al., 2001). Cognitively preserved controls underwent full neuropsychological assessment 18 and 54 months post-inclusion. In order to consider both decrement and improvement of performances in a wide range of cognitive functions, we defined a continuous cognitive score (CCS) after converting all neuropsychological values to *z* scores. Subsequently, the number of cognitive tests with improved performances at follow-up (at least 0.5 standard deviation (SD) higher compared to inclusion) was established (range, 0–14). The same approach yielded the number of tests with decreased performances (range, 0–14). The final continuous cognitive score (CCS) represents the number of tests with improved minus the number of tests with decreased performances. Change in cognition between inclusion and last follow-up was defined as the sum of the continuous cognitive scores at 18 and 54 months (Montandon et al., 2020b).

### APOE Epsilon 4 Status

APOE epsilon 4 status was assessed as described earlier (Zanchi et al., 2017b). Subjects were divided according to the APOE epsilon 4 allele presence (4/3 vs. 3/3, 3/2 carriers).

### MR Imaging

At baseline, imaging data were acquired on a 3T MRI scanner (TRIO SIEMENS Medical Systems, Erlangen, Germany). The structural high-resolution T1-weighted anatomical scan was performed with the following fundamental parameters: 256 × 256 matrix, 176 slices, 1 mm isotropic, TR = 2,300 ms, TE 2.27 ms; axial T2w sequences: 512 × 310 matrix, 30 slices, 4 mm thickness, TR 4,000 ms, TE 105 ms; susceptibility-weighted imaging (SWI): 256 × 208 matrix, 128 slices, TR 28 ms, TE 20 ms); pulsed ASL: 64 × 64 matrix, 20 slices, 6 mm thickness, TR 4,000 ms, TE 12 ms, inversion time 1,800 ms. At follow-up, data were acquired on a 3T MR750w scanner (GE Healthcare, Milwaukee, Wisconsin), including a high-resolution anatomical 3DT1: 254 × 254 matrix, 178 slices, 1 mm isotropic, TR 7.2 ms; axial T2w sequences: 512 × 512 matrix, 30 slices, 4 mm thickness, TR 6,900 ms, TE 105 ms; susceptibility-weighted angiography (SWAN): 320 × 288 matrix, 176 slices, TR 28 ms, TE 20 ms; multi-delay (7) pseudo-continuous ASL (PCASL) 128 × 128 matrix, 32 slices, 4 mm thickness, TR 5,936 ms, TE 10.5 ms, post label delays 1.00, 1.22, 1.48, 1.78, 2.15, 2.62, and 3.32 s.

### Amyloid PET Imaging

Fifty-nine ^18^F-Florbetapir- (Amyvid) and 31 ^18^F-Flutemetamol-PET (Vizamyl) data were obtained using Siemens BiographTM mCT and GE Healthcare Discovery PET/CT 710 scanners of varying resolution and following different platform-specific acquisition protocols. The ^18^F-Florbetapir images were acquired 50–70 min after injection and the ^18^F-Flutemetanol images 90–120 min after injection. PET images were reconstructed according to the ADNI protocol in order to increase data uniformity across the multicenter acquisitions. More information on the different imaging protocols for PET acquisition can be found on the ADNI website^3^.

### FDG-PET Imaging

PET/CT data acquisition was performed on a Siemens Biograph^TM^ mCT or Vision scanner according to the guidelines of the European Association of Nuclear Medicine (EANM; Varrone et al., 2009). The PET acquisition was started 30 min after injection of 200 MBq of ^18^F-FDG. The PET emission study (20 min, one-bed position) followed immediately the CT study used for attenuation correction. Ultra-law dose brain CT imaging was performed under standard conditions (120 kVp, 20 mAs, 128 × 0.6 collimation, a pitch of 1 and 1 s per rotation).

### MRI Analysis

#### Visual MRI Assessment

A board-certified specialist in neuroradiology (SH) blind to the CCS data was in charge of the MRI analysis. MTA was assessed at baseline according to the established score (Scheltens et al., 1995), from 0 (no atrophy) to 4 (significant atrophy). At follow-up, MTA change was graded from 0 to 3 (0 = stable, 1 = slight increase, 2 = moderate increase, 3 = strong increase). A score of 1 indicates that the MTA score remains the same, yet there is a slight increase in the visual perception of MTA atrophy. Scores of 2 correspond to a 1 grade progression of the MTA score. WMH load at baseline was assessed according to the Fazekas score (Fazekas et al., 1987) ranging from 0 (no white matter lesions) to 3 (confluent white matter lesions). At follow-up, the equivalent score of 0–3 was used (0 = stable, 1 = slight increase, 2 = moderate increase, 3 = strong increase) using the same interpretation scheme. CMB was identified on the SWI or SWAN sequences. The corresponding phase images were also analyzed to discriminate probable CMB vs. micro-calcifications (Haller et al., 2018). At baseline, the total number of CMB and number of CMB per location (supratentorial superficial, supratentorial deep, and infratentorial) were assessed at baseline as well as their increase at follow-up.

#### ROI MRI Assessment

3DT1 MRIs were preprocessed with the FSL software package^4^, according to the standard procedure. The essential processing steps included brain extraction with the FSL Brain Extraction Tool^5^, tissue-type segmentation with the FMRIB Automated Segmentation Tool^6^, nonlinear transformation into Montreal Neurological Institute reference space, and creation of a study-specific GM template to which the native GM images were then nonlinearly reregistered. The modulated segmented images were then smoothed with an isotropic Gaussian kernel with a width of 2 mm. Furthermore, we created a mask from the Harvard-Oxford atlas (Desikan et al., 2006) for the bilateral medial temporal cortex, hippocampus, amygdala, parietal and posterior cingulate cortex (PCC; AD-signature areas) as well as the occipital lobe (as control area) that was then applied to the GM image of the study-specific template, and we obtained GM density values (mean signal of ROI) in each area of interest.

### Amyloid PET Analysis

All amyloid PET images were analyzed by an independent, board-certified specialist in nuclear medicine (VG) without previous knowledge of the neuropsychological data, according to the tracer-specific standardized operating procedures approved by the European Medicinal Agency. Regions of interest included the lateral frontal, parietal, posterior cingulate and precuneus, anterior cingulate, and medial temporal regions in either of the two hemispheres (AD-signature areas) as well as striatal nuclei (as control area; Buckley et al., 2017). In order to assess the global amyloid burden in the present series, we also calculated cortical SUVR values as provided by the centiloid project with pons as a reference region (Klunk et al., 2015). Cortical uptake was calculated using the regions from the Harvard-Oxford atlas (Desikan et al., 2006).

### FDG-PET Analysis

FDG-PET reading was performed visually using an automated voxel-wise comparison with a reference database (Varrone et al., 2009). Images were classified as pathological when significant reductions of regional glucose metabolism were observed compared to the reference. FDG-PET images were quantitatively analyzed using BRASS automated functional brain analysis software (HERMES BRASS software, Nuclear Diagnostic AB, Sweden). Briefly, BRASS fits and compares patients’ images to 3D reference templates created from images of healthy subjects (Slomka et al., 2001). FDG-PET images were warped (Radau et al., 2001) individually to the BRASS template and we extracted the mean activity normalized by the global brain activity in the standard set of 63 anatomical ROIs provided by the software. As Hermes BRASS is a commercial software, with its own reference regions, we matched the regions of FDG-PET with the Harvard-Oxford atlas used for VBM and amyloid PET imaging.

For the analysis of FDG data, similar ROIs to amyloid PET assessment were used.

### Statistical Analysis

Comparisons among the three education attainment levels were performed with Cuzick nonparametric test for trend across ordered groups, Kruskal-Wallis, or oneway ANOVA depending on the variable’s distribution. Three types of clinical endpoints were considered in the present analysis. The primary endpoint was the change of the continuous cognitive score upon follow-up. An additional endpoint (closer to clinical reality) was the improvement in the continuous cognitive score upon follow-up. Finally and in order to explore the association between imaging parameters and cognitive domains (instead of global cognition), we used *z* score differences in neuropsychological tests (Digit span and Corsi visual memory span forward for working memory, Shape test for visual episodic memory, phonemic verbal fluency and Trail Making B for executive functions, and Digit Symbol Coding for attention) as additional endpoints in regression models. Multiple linear regression models were built to search for independent variables associated with longitudinal changes in cognitive scores (or z-score changes in cognitive functions) expressed as a continuous dependent variable. In addition, multiple logistic regression models were used to examine the independent variables associated with a binary cognitive variable. The binary variable was defined as follows: negative cognitive score changes (cognitive decrement) were coded as 0 and positive and null cognitive score changes were coded as 1. Likelihood-ratio tests were used to compare regression models with qualitative (Model 1) vs. qualitative plus quantitative (Model 2) imaging variables. Corresponding areas under the receiver operating characteristic curve (AUC) along with their 95% confidence interval (95% CI) were compared using the “roccomp” Stata’s command. P values lower than 0.05 were considered significant. Stata version 16.1 (Stata Corp., College Station, TX) was used for analyses.

## Results

The final series included 90 participants (55 female, 61.1%). The mean age was 79.2 ± 3.7 years with an average MMSE at baseline of 28.6 ± 1.0. The mean CCS change was −0.5 ± 3.7. Among the present cases 39 (43.3%) worsened, 14 (15.6%) remained unchanged and 37 (41.1%) improved. Fifteen cases were APOE4 carriers (16.7%).

Table 1 summarizes imaging differences as a function of the level of formal education in the present series. A lower level of education was associated with the female sex but not with the worst cognitive evolution, APOE4 genotype, and MMSE scores at baseline. There were no education-related significant differences in imaging variables. Education and APOE4 were not related to the CCS change over time. Importantly, 82% of cases had subthreshold amyloid deposits defined as mean distribution volume ratio of amyloid lower than 1.07 or 1.3 independently of the visual inspection (Veitch et al., 2019). Table 2 provides an overview of imaging differences according to the clinical evolution (cognitive decrement vs. improvement and null).

**Table 2:** Demographic, clinical and imaging data by clinical evolution (cognitive worsening vs. unchanged or improved).

	Binary cognitive outcome			
	Worsened	Unchanged or improved	Total	*P*-Values
N	39	51	90	
MRI				
Number of microbleeds				0.652
0	25 (64.1%)	33 (64.7%)	58 (64.4%)
1	6 (15.4%)	9 (17.6%)	15 (16.7%)
2+	8 (20.5%)	9 (17.6%)	17 (18.9%)
Fazekas score				0.684
Absent	14 (35.9%)	21 (41.2%)	35 (38.9%)
Mild	20 (51.3%)	16 (31.4%)	36 (40.0%)
Moderate	4 (10.3%)	11 (21.6%)	15 (16.7%)
Severe	1 (2.6%)	3 (5.9%)	4 (4.4%)
MTA right				0.680
No atrophy	8 (20.5%)	15 (29.4%)	23 (25.6%)
Only widening of choroid fissure	21 (53.8%)	21 (41.2%)	42 (46.7%)
Also widening of temporal horn of lat. ventricle	8 (20.5%)	15 (29.4%)	23 (25.6%)
Moderate loss of hippocampal volume	2 (5.1%)	0 (0.0%)	2 (2.2%)	
MTA left				0.728
No atrophy	9 (23.1%)	13 (25.5%)	22 (24.4%)
Only widening of choroid fissure	22 (56.4%)	29 (56.9%)	51 (56.7%)
Also widening of temporal horn of lat. ventricle	7 (17.9%)	8 (15.7%)	15 (16.7%)
Moderate loss of hippocampal volume	1 (2.6%)	1 (2.0%)	2 (2.2%)
Posterior cingulate MRI volume change *100	−2.0 ± 1.7	−1.5 ± 1.7	−1.7± 1.7	0.163
Baseline hippocampus MRI volume	0.4 ± 0.0	0.4 ± 0.0	0.4 ± 0.0	0.641
PET		
Amy PET positive	11 (28.2%)	11 (21.6%)	22 (24.4%)	0.464
FDG PET abnormal	9 (23.1%)	13 (25.5%)	22 (24.4%)	0.464
Vermis FDG-PET	1583.4 ± 194.0	1509.9 ± 225.3	1541.7 ± 214.3	0.100
Mesial temporal cortex FDG-PET	1705.7 ± 195.5	1697.3 ± 237.8	1700.9 ± 219.3	0.854
Superior temporal cortex FDG-PET	1692.0 ± 196.3	1662.9 ± 231.7	1675.5 ± 216.4	0.521

In multivariable models, the presence of more or equal to four CMB at inclusion and slight atrophy of the right medial temporal lobe (MTL) at follow-up were independently related to the CCS explaining 6% of its cognitive variability. Importantly, Fazekas score assessment of WMH, as well as amyloid positivity and abnormal FDG-PET were not retained as significant variables in these models. When the ROI data were added, volume loss in PCC, lower baseline hippocampus volume, and lower MTL metabolism were all associated with CCS decrement. In a multivariable model including both qualitative and quantitative imaging data, the percentage of CCS variability explained by the model increased to 24.5%. The addition of a quantitative imaging marker significantly increases the predictive power of the regression model as documented by the likelihood-ratio test (*p* < 0.0001; Table 3). None of the other ROI regional amyloid or FDG-PET values were retained in these models.

**Table 3:** Continuous cognitive score difference explained by multiple linear regressions with qualitative (Model 1) vs. qualitative plus quantitative (Model 2) imaging variables.

	Model 1	Model 2
	Coeff (95% CI)	Coeff (95% CI)	
Qualitative
Number of microbleeds
0	0.000 (0.000, 0.000)	0.000 (0.000, 0.000)
1	−0.970 (− 3.072, 1.132)	−1.428 (−3.394, 0.537)
2+	−0.796 (− 2.818, 1.226)	−1.069 (− 2.900, 0.762)
MTA right
No atrophy	0.000 (0.000, 0.000)	0.000 (0.000, 0.000)
Only widening of choroid fissure	−1.842 (−3.539, −0.145)*	−0.804 (−2.431, 0.824)
Also widening of temporal horn of lat. ventricle	−0.197 (− 2.577, 2.183)	1.184 (− 1.073, 3.442)
Moderate loss of hippocampal volume	−0.725 (−5.109, 3.659)	−2.567 (−6.940, 1.806)
Quantitative
Posterior cingulate MRI volume change *100		0.518 (0.095, 0.940)*
Baseline hippocampus MRI volume		26.956 (4.667, 49.246)*
Vermis FDG-PET		−0.007 (−0.013, −0.002)*
Mesial temporal cortex FDG-PET		0.021 (0.009, 0.034)*
Superior temporal cortex FDG-PET		−0.018 (−0.029, −0.006)*

*Model 1 includes CMB numbers and visual assessment of MTA whereas Model 2 takes also into account volumetric MRI changes in the posterior cingulate cortex, baseline hippocampal volume, and mesial temporal cortex metabolism. Models are compared with likelihood-ratio test: *p* < 0.0001*. *Indicates significant values.

**Figure 1:**
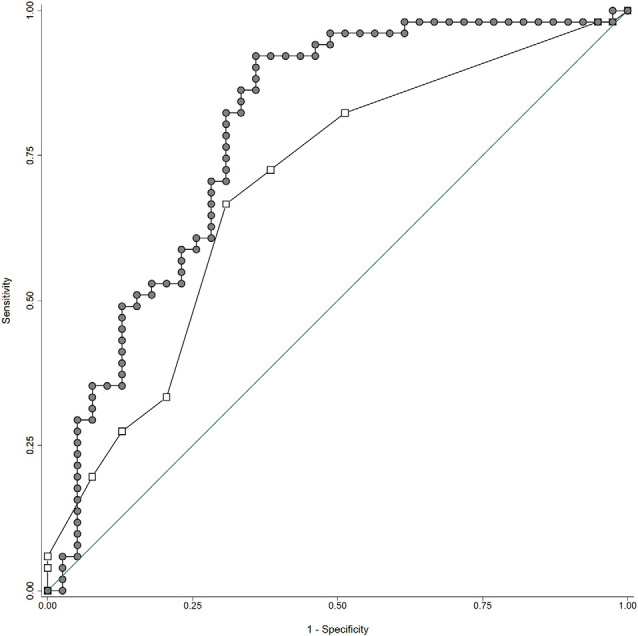
ROC curves for qualitative (continuous line: area under the curve of 0.69) vs. mixed imaging data (area under the curve of 0.79). Note that the addition of quantitative imaging parameters significantly improves the accuracy of the regression models in predicting the cognitive outcome (worsened vs. unchanged or improved). See text for details.

In order to be closer to the clinical reality, we also built multiple logistic regression models with binary assessment of the cognitive trajectory based on the CCS (improved or stable vs. deteriorated at follow-up; Table 4). CMB ≥ 4 at inclusion and subtle right MTA at follow-up were still related to the cognitive outcome. The addition of ROI data revealed an independent association between volume loss in PCC as well as lower MTL metabolism (but not other ROI regional data) and cognitive deterioration. The area under the ROC curve increased from 0.69 for the first to 0.79 for the second model (Figure 1; Chi2 = 4.89; *P* = 0.0269).

**Table 4:** Binary evolution of the continuous cognitive score (improvement and null) explained by multiple logistic regressions with qualitative (Model 1) vs. qualitative plus quantitative (Model 2) imaging variables.

	Model 1		Model 2	
	OR (95% CI)	p	OR (95% CI)	p
Qualitative
Number of microbleeds
0	1.00 (1.00, 1.00)		1.00 (1.00, 1.00)
1	1.18 (0.34, 4.09)	0.793	1.14 (0.29, 4.48)	0.848
2–3	1.02 (0.32, 3.29)	0.972	1.00 (0.27, 3.68)	0.997
4–6	−4.50 (−9.00, −0.71)	0.035*	−4.15 (− 7.40, −0.89)	0.026*
MTA right
No atrophy	1.00 (1.00, 1.00)		1.00 (1.00, 1.00)
Only widening of choroid fissure	0.24 (0.09, 0.65)	0.005*	0.27 (0.09, 0.85)	0.025*
Also widening of temporal horn of lat. ventricle	1.26 (0.28, 5.68)	0.765	1.94 (0.37, 10.02)	0.430
Moderate loss of hippocampal volume	0.18 (0.01, 2.31)	0.190	0.03 (0.00, 0.90)	0.043 *
Quantitative
Posterior cingulate MRI volume change *100			1.40 (1.02, 1.91)	0.036 *
Baseline hippocampus MRI volume			79.30 (0.00, 4.3 10^8^)	0.580
Mesial temporal cortex FDG-PET			1.01 (1.00, 1.02)	0.035*

*Models are compared with likelihood-ratio test: *p* = 0.0310*. *Indicates significant values.

In order to address the relationship between imaging data and cognitive domains, we also built multiple linear regression models using *z* scores differences for selected cognitive functions as dependent variable (digit span and Corsi visual memory span forward for working memory, Shape test for visual episodic memory, phonemic verbal fluency and Trail Making B for executive functions, and Digit Symbol Coding for attention). Among them, Trail Making Test B *z* score change was associated with the presence of more or equal to four CMB at inclusion (regression coefficient: −8914, CI: −14758, −3070) and severe right MTA (regression coefficient: −16392, CI: −30351, −2434) at follow-up (8.3% of its variability). The Shape test *z* score change was also related to the presence of more or equal to four CMB at inclusion (regression coefficient: −1.859, CI: −3.679, −0.039; 4.6% of its variability). No significant association between the remaining *z* score changes and imaging data was found.

## Discussion

The present data reveal that the inclusion of quantitative imaging data significantly improves clinico-radiological correlations in elderly controls compared to the single consideration of visual inspection. Moreover, they show that MRI volumetry in PCC and FDG-PET metabolism in MTL, but not regional amyloid load, are significantly associated with the worst neuropsychological performances at this very early stage of the neurodegenerative process. Of note, these differences are found only in multivariable analyses taking into account the interdependence between the different imaging modalities.

Confirming the data of our previous study, CMB number higher than 4 at baseline and the increase of visually assessed right MTA, a well-known AD-signature marker, at follow-up are independently associated with decreased CCS. The negative relationship between CMB number and cognition in healthy controls parallels the findings from three population-based studies [Rotterdam (Akoudad et al., 2016), Framingham Heart (Romero et al., 2017); and AGES-Reyjkavik (Ding et al., 2017)] in cases free from dementia. The percentage of CCS variability explained by this parameter was slightly lower than in our previous study (6% vs. 10%; 20) that did not include quantitative imaging data. In addition, MTA worsening impacts on cognition in this study (Montandon et al., 2020a). As previously suggested, only the right MTA increase was related to the cognitive outcome in regression models including both qualitative and mixed imaging markers (Frisoni et al., 2007; Shen et al., 2011; Cavedo et al., 2014). Importantly, these parameters were also related with *z* score changes of visual episodic memory (Shape test) and executive function (Trail Making B test) further supporting their relevance in clinico-radiological correlations in healthy controls.

In contrast, the amyloid load was not related to decreased cognitive performances in this sample not only in qualitative models but also when regional scores are taken into account. Significant amyloid deposition was initially thought to correlate with the longitudinal decrement of cognition (Petersen et al., 2016). More recent observations challenged this viewpoint showing that the association between Aβ and cognition mostly concerns cases with a lower amyloid burden (Knopman et al., 2018). Longitudinal studies failed to detect an association between amyloid load and neuropsychological performances in healthy controls and MCI cases (Dubois et al., 2018). The present findings are consistent with these results and suggest that amyloid deposition is not a valid imaging marker of cognitive decrement in our sample. This was also the case for WMH assessed semi-quantitatively with the Fazekas score and abnormal FDG-PET (Devanand et al., 2010; Alber et al., 2019).

A different pattern of clinico-radiological correlations emerges when quantitative data were included in the regression models. MTL hypometabolism and PCC volume loss were independently associated with the cognitive outcome both in regression models using CCS as a continuous or binary variable. Hippocampal volume at inclusion was significantly associated with CCS but this association, already well established in MCI cases (Ottoy et al., 2019; Guo et al., 2020; Zeng et al., 2020), did not persist in binary models indicating that its assessment in elderly controls should be reserved in sophisticated clinical settings where a quantitative assessment of cognitive functions is available. The key role of MTL hypometabolism in cognitive decrement is not surprising but was mainly thought to concern MCI transition to AD (Mosconi, 2005). Of note and contrasting with previous observations in MCI and AD cases (Mosconi et al., 2006), visual rating of this parameter was not related to the cognitive outcome in the present series indicating that the use of quantitative tools is needed in healthy controls to detect the impact of decreased MTL metabolism on subtle cognitive changes. In the same line, PCC volume loss was most frequently reported in MCI and mainly in clinically overt AD (Shiino et al., 2006; Choo et al., 2010; Gili et al., 2011; Teipel et al., 2016). Our results confirm the observations of Pengas et al. (2010) reporting that a significant atrophy of PCC takes place in incipient AD cases suggesting that PPC volumetry may be a useful and early marker of neurodegeneration long before the emergence of cognitive deficits in the course of brain aging.

The present data are also relevant in the current debate about the added value of the inclusion of quantitative imaging among AD biomarkers. In our series, the addition of MTL metabolic and PPC volumetric data significantly increases the percentage of cognitive variance explained by visual inspection from 6% to 24.5%. When binary CCS outcome was considered, the AUC values of ROC curves for quantitative and mixed imaging data also progressed significantly from 0.69 to 0.79. Although these increases may seem modest when considering the extra cost of ROI analysis one should keep in mind that our cases are cognitively preserved at baseline with only subtle neuropsychological changes within the normal age-adjusted range at follow-up. The association of quantitative imaging markers with cognition is fatally harder to establish in this particular context (Lauriola et al., 2017; Perrotin et al., 2017). Most importantly, our results demonstrate that only PPC volumetry and MTL hypometabolism are retained in multivariable models taking into account the interdependence of functional and structural alterations in AD-signature cortical areas. From a cost-efficiency viewpoint, these findings allow for identifying a restricted panel of imaging markers that have clinical relevance at the very early stages of brain aging including visually inspected lobar CMB, MTL progressive atrophy, and ROI analysis of PCC volume loss and MTL hypometabolism.

Among the strengths of the present study, one can note the inclusion of several imaging modalities exploring both lesion burden (amyloid deposition, WMH, and CMB), and subsequent changes in brain structure and metabolism (MTA, abnormal PET), detailed cognitive analysis and, most importantly use of multivariable models including sequentially qualitative and quantitative imaging data. Several limitations should be considered. The present series includes highly educated elders with a mean age close to 80 years and no medical comorbidities that are certainly not representative of the whole spectrum of brain aging. Our observations should thus be interpreted with caution in terms of generalizability. In order to save statistical power, we limited our ROI analyses in selected AD-signature cortical areas and cannot comment on the clinical relevance of automated processing tools. Unlike FDG-PET, both for amyloid PET and VBM MRI analyses, no widely used clinical software are available. The definition of anatomical areas using the BRASS commercial software for FDG-PET may fatally not overlap with that of the Harvard-Oxford atlas used for VBM and amyloid PET imaging. The criterion used here to define CCS changes (−0.5 SD compared to inclusion) remains arbitrary. Although our approach has the advantage to take into consideration a wide range of cognitive functions and in the absence of longer follow-up, we cannot exclude that our observations are threshold-dependent. An additional limitation concerns the absence of tau and translocator protein (a well-established marker of neuroinflammation) imaging to complete the AD-signature characterization. In fact, two recent studies reported that early tau accumulation takes place in elderly controls in the absence of significant amyloid burden, Das et al. reported that tau burden correlated with both mesial temporal lobe atrophy and poorer memory performance in amyloid negative cases (Das et al., 2019). In the same line, an increased tau uptake was reported in bilateral temporal and retrosplenial cortex of amyloid negative cases and was related to significant atrophy in the same regions (Harrison et al., 2019). Moreover, an association between translocator protein and amyloid PET binding was reported at the very early stages of amyloid pathology in asymptomatic elders (Toppala et al., 2021). Last but not least, MRI imaging was performed twice during the 4.5-year period. It is likely that some of our cases display age-related physiological decline whereas some of them are already incipient AD cases. Importantly, the vast majority of our cases had subthreshold amyloid deposits that are now thought to be associated with decreased memory performances, increased tau uptake, and mesial temporal lobe atrophy in longer follow-ups (Landau et al., 2018; Das et al., 2019). Additional time points with multiple amyloid assessments are needed to explore the validity of quantitative imaging data in the prediction of cognitive fate in asymptomatic elders.

## Data Availability Statement

The raw data supporting the conclusions of this article will be made available by the authors, without undue reservation.

## Ethics Statement

The studies involving human participants were reviewed and approved by Ethics Committee of the Geneva University Hospitals. The patients/participants provided their written informed consent to participate in this study.

## Author Contributions

Conceived the study: PG and FRH. Recruitment: CR, M-LM, SH, and VG. Neuropsychology supervising: M-LM, CR, and PG. Imaging: SH, M-LM, and VG. Data preparation: M-LM and FRH. Analyzed the data: M-LM, FRH, SH, VG, and PG. Manuscript writing: PG, M-LM, FRH, SH, VG, and CR. All authors contributed to the article and approved the submitted version.

## Conflict of Interest

The authors declare that the research was conducted in the absence of any commercial or financial relationships that could be construed as a potential conflict of interest.

## Footnotes

^1^http://www.fmrib.ox.ac.uk/fsl/

^2^https://surfer.nmr.mgh.harvard.edu

^3^https://adni.loni.usc.edu/methods/

^4^http://www.fmrib.ox.ac.uk/fsl/

^5^http://fsl.fmrib.ox.ac.uk/fsl/fslwiki/BET

^6^http://fsl.fmrib.ox.ac.uk/fsl/fslwiki/fast
